# Tumor microenvironment in giant cell tumor of bone: evaluation of PD-L1 expression and SIRPα infiltration after denosumab treatment

**DOI:** 10.1038/s41598-021-94022-w

**Published:** 2021-07-20

**Authors:** Yu Toda, Kenichi Kohashi, Hidetaka Yamamoto, Shin Ishihara, Yoshihiro Ito, Yosuke Susuki, Kengo Kawaguchi, Daisuke Kiyozawa, Dai Takamatsu, Izumi Kinoshita, Yuichi Yamada, Junki Maehara, Atsushi Kimura, Sadafumi Tamiya, Kenichi Taguchi, Tomoya Matsunobu, Yoshihiro Matsumoto, Yasuharu Nakashima, Masaaki Mawatari, Yoshinao Oda

**Affiliations:** 1 Department of Anatomic Pathology, Pathological Sciences, Graduate School of Medical Sciences, Kyushu University, Maidashi 3-1-1, Higashi-ku, Fukuoka, 812-8582 Japan; 2 Department of Clinical Radiology, Graduate School of Medical Sciences, Kyushu University, Fukuoka, Japan; 3 Department of Orthopaedic Surgery, Graduate School of Medical Sciences, Kyushu University, Fukuoka, Japan; 4 Department of Pathology, Kitakyushu Municipal Medical Center, Fukuoka, Japan; 5 Department of Pathology, National Hospital Organization Kyushu Cancer Center, Fukuoka, Japan; 6 Department of Orthopaedic Surgery, Kyushu Rosai Hospital, Fukuoka, Japan; 7 Department of Orthopaedic Surgery, Faculty of Medicine, Saga University, Saga, Japan

**Keywords:** Bone cancer, Cancer microenvironment, Cancer immunotherapy

## Abstract

Giant cell tumor of bone (GCTB) is an intermediate malignant bone tumor that is locally aggressive and rarely metastasizes. Denosumab, which is a receptor activator of nuclear factor kappa B ligand (RANKL) inhibitor, can be used to treat GCTB. We focused on potential immunotherapy for GCTB and investigated the tumor microenvironment of GCTB. Programmed death-ligand 1 (PD-L1) and indoleamine 2,3-dioxygenase 1 (IDO1) expression and signal-regulatory protein alpha (SIRPα), forkhead box P3 (FOXP3), and cluster of differentiation 8 (CD8) infiltration were assessed by immunohistochemical studies of 137 tumor tissues from 96 patients. Of the naive primary specimens, 28% exhibited PD-L1 expression and 39% exhibited IDO1 expression. There was significantly more SIRPα^+^, FOXP3^+^, and CD8^+^ cell infiltration in PD-L1- and IDO1-positive tumors than in PD-L1- and IDO1-negative tumors. The frequency of PD-L1 expression and SIRPα^+^ cell infiltration in recurrent lesions treated with denosumab was significantly higher than in primary lesions and recurrent lesions not treated with denosumab. PD-L1 expression and higher SIRPα^+^ cell infiltration were significantly correlated with shorter recurrence-free survival. PD-L1 and SIRPα immune checkpoint inhibitors may provide clinical benefit in GCTB patients with recurrent lesions after denosumab therapy.

## Introduction

Giant cell tumor of bone (GCTB) is an intermediate malignant bone tumor with frequent local recurrence and rare metastasis^1^. GCTB typically arises in the metaphysis and epiphysis of long bones and in the spine between the ages of 20 and 40 years^2–5^. Histologically, GCTB is composed of oval- and short spindle-shaped tumor cells, mesenchymal stromal cells, mononuclear monocytes, and osteoclast-like multinucleated giant cells^1,4,6^. Mesenchymal stromal tumor cells and osteoclast-like multinucleated giant cells overexpress receptor activator of nuclear factor kappa B (RANK) and receptor activator of nuclear factor kappa B ligand (RANKL), respectively. *H3F3A* mutations occur in 90% of GCTB cases, and conventional GCTB generally harbors p.G34W mutations^4,7–11^. Denosumab is a human monoclonal antibody that inhibits the receptor activation of RANKL and the RANKL pathway^11–13^. In Japan in 2014, denosumab was approved for the treatment of unresectable and recurrent GCTB^14^. However, denosumab-treated GCTB has recurrence potential. Healey et al. suggested that the risk of malignant transformation with denosumab is increased^15^. The main objective of recent studies has been to investigate potential immunotherapy against GCTB. Programmed death-ligand 1 (PD-L1) is the ligand of programmed cell death protein 1 (PD-1), and it is thought to promote evasion of the antitumor immune response by suppressing T-cell function. Several investigations and clinical trials concerning the PD-L1/PD-1 axis have been developed for various cancers, including malignant bone tumors^16–21^. Metovic et al. investigated PD-L1 expression in GCTB patients and reported that PD-L1 expression was correlated with shorter disease-free survival^22^.

Indoleamine 2,3-dioxygenase 1 (IDO1) is an enzyme of tryptophan metabolism, and it is associated with poor prognosis by enabling malignant tumors to avoid immune surveillance^12^. The expression of PD-L1 and IDO1 and the clinicopathological impact of PD-L1 and IDO1 co-expression have recently been investigated in several malignant tumors, such as lung cancer^23–28^, renal cell carcinoma^29^, thyroid cancer^30^, and osteosarcoma^20^.

Cluster of differentiation 47 (CD47) and signal-regulatory protein alpha (SIRPα) are “don’t eat me” signals, and they promote escape from phagocytosis in malignant tumors^31–33^, enabling lymphoma cells to evade phagocytosis and thereby promoting tumor growth^32^. Dancsok et al. investigated SIRPα expression in various bone and soft tissue sarcomas and reported that some sarcomas showed shorter progression-free survival with PD-L1 expression^31^.

We retrospectively analyzed PD-L1 and IDO1 expression and SIRPα, cluster of differentiation 8 (CD8), and forkhead box P3 (FOXP3) immune cell infiltration, and we examined their effects on the clinicopathological parameters of GCTB and their prognostic value in GCTB.

## Results

### Clinical results

The clinicopathological features of the subjects are shown in Table 1. The median age at initial diagnosis was 33 (17–84) years. The subjects included 54 females and 42 males. The tumors were mainly located in the femur or tibia (Table 1). Four patients did not undergo surgery and were treated only with denosumab. Ten patients received denosumab (six for neoadjuvant therapy and four for recurrence). Recurrence- and metastasis-free survival data were available for 78 patients, with follow-up ranging from 1 to 332 months (median: 58 months). In this study, 18 patients (23%) had local recurrence and eight (10%) had distant metastasis. All patients with metastasis developed pulmonary metastases. There were no patients with tumor-related death.

**Table1:** Clinico-radio-pathological features.

Parameter (N = 96)	No. of patients	%
**Clinical features**		
Median age: (mean, median, range): 37, 33, 17–84 (years old)		
Gender		
Male	42	43.8
Female	54	56.3
Tumor site		
Femur	32	33.3
Tibia	25	26.0
Radius	10	10.4
Spine	8	8.3
Fibula	8	8.3
Humerus	6	6.3
Pelvic bone	3	3.1
Short bone	2	2.1
Rib	1	1.0
Ulna	1	1.0
Size (mean, median, range): 4.3, 4.2, 0.6–79 (mm)		
< 5 cm	41	42.7
≥ 5 cm	27	28.1
No data	28	29.2
Follow-up (N = 78) (mean, median, range): 75, 58, 1–332 (months)		
Local recurrence		
Present (mean, median, range): 37, 17, 2–198 (months)	18	23.1
Absent	60	76.9
Distant metastasis (lung)		
Present (mean, median, range): 64, 21, 0–289 (months)	8	10.3
Absent	70	89.7
**Imaging findings**		
Pathological fracture		
Absent	70	72.9
Present	6	6.3
No data	20	20.8
**Treatment**		
Initial surgery		
Given	92	95.8
Curettage	60	65.2
Wide resection or Total en bloc spondylectomy	14	15.2
No data	18	29.2
Not given	4	4.2
Denosumab		
Neo adjuvant	6	6.3
After Recurrence	4	4.2
Not given	86	89.6
**Genetic features**		
H3F3A mutation		
G34W	90	93.8
G34R	5	5.2
G34V	1	1.0

### Radiological features

Radiological images, including plain radiographs, computed tomography scans, and/or magnetic resonance imaging scans, or medical records were available for 76 patients. Maximum diameters (N = 68) and pathological fractures (N = 76) were investigated. The radiological characteristics are shown in Table 1. Radiologically, the median of the maximum diameters was 4.3 cm (range: 0.6–7.9 cm). A pathological fracture was apparent in six of 76 cases (8%).

### Immunohistochemistry

#### Immunoexpression in primary tumors treated without denosumab

##### Association of PD-L1 and IDO1 expression and SIRPα^+^ macrophage and FOXP3^+^ and CD8^+^ lymphocyte infiltration with clinicopathological features

**Figure 1:**
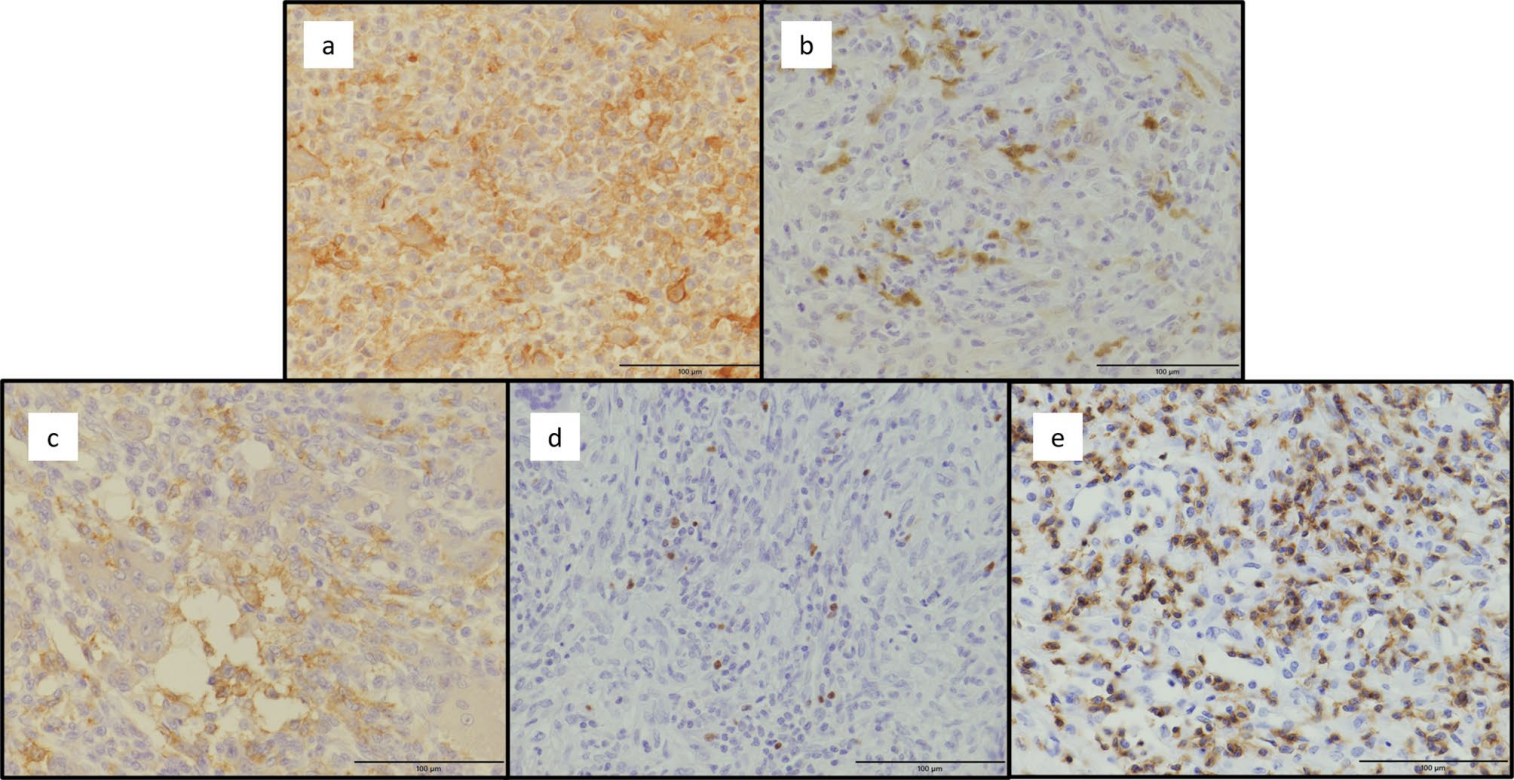
Representative images of the immunoexpression of PD-L1 (**a**), IDO1 (**b**), SIRPα (**c**), FOXP3 (**d**) and CD8 (**e**) in GCTB. PD-L1, SIRPα and CD8: Membranous staining, IDO1: Cytoplasmic and membranous staining. FOXP3: Nuclear staining Scale bars: 100 µm. PD-L1: programmed death ligand 1, IDO1: indoleamine 2,3-dioxygenase 1, SIRPα: Signal-regulatory protein alpha, FOXP3: forkhead box P3, CD8: cluster of differentiation 8.

Representative images of the PD-L1, IDO1, SIRPα, FOXP3, and CD8 immunohistochemical studies are shown in Fig. 1a–e. Among the 92 non-denosumab-treated primary specimens, 26 (28%) exhibited PD-L1 expression and 36 (39%) exhibited IDO1 expression (Table 2). PD-L1 expression was more frequent in the cases that underwent neoadjuvant denosumab therapy than in the cases that did not (*P* = 0.0064) (Table 2). IDO1 expression was more frequent in cases with GCTB in the extremity than in cases with GCTB in the trunk (*P* = 0.0247) (Table 2). Frequent mitotic figures (≥ 10/10 HPF) showed significant correlation with FOXP3 infiltration. Histologically, spindle cell features were correlated with SIRPα infiltration (Table 2).

**Table 2:** The correlations of clinicopathological characteristics with immunoexpression of PD-L1 and IDO1 and immunoexpression of infiltration of SIRPα, FOXP3 and CD8 positive cells in primary naive specimens.

		PD-L1 − (%)	PD-L1 + (%)	*P* value	IDO1 − (%)	IDO1 + (%)	*P* value	SIRPα-low (%)	SIRPα-high (%)	*P* value	FOXP3-low (%)	FOXP3-high (%)	*P* value	CD8-low (%)	CD8-high (%)	*P* value
All		66 (72)	26 (28)		56 (61)	36 (39)		47 (51)	45 (49)		44 (49)	46 (51)		46 (51)	44 (49)	
**Clinical and imaging features**																
Age, y	< 33	35 (38)	14 (15)	1.0000	29 (32)	20 (22)	0.8312	27 (29)	22 (24)	0.5309	25 (28)	22 (24)	0.4082	28 (31)	19 (21)	0.1391
	≥ 33	31 (34)	12 (13)		27 (29)	16 (17)		20 (22)	23 (25)		19 (21)	24 (27)		18 (20)	25 (28)	
Gender	Male	27 (29)	13 (14)	0.4877	21 (23)	19 (21)	0.1967	16 (17)	24 (26)	0.0920	13 (14)	26 (29)	**0.0116**	14 (16)	25 (28)	0.0188
	Female	39 (42)	13 (14)		35 (38)	17 (18)		31 (34)	21 (23)		31 (34)	20 (22)		32 (36)	19 (21)	
Tumor site	Extremity	56 (61)	24 (26)	0.4981	45 (49)	35 (38)	**0.0247**	39 (42)	41 (45)	0.3553	34 (38)	44 (49)	**0.0130**	39 (43)	39 (43)	0.7586
	Axial skeleton	10 (11)	2 (2)		11 (12)	1 (1)		8 (9)	4 (4)		10 (11)	2 (2)		7 (8)	5 (6)	
Extremity	Upper	10 (12)	7 (9)	0.3708	8 (10)	9 (11)	0.4208	10 (13)	7 (9)	0.4182	25 (32)	35 (45)	0.7839	11 (14)	6 (8)	0.2725
	Lower	46 (58)	10 (13)		37 (46)	26 (33)		29 (36)	34 (43)		8 (10)	9 (12)		28 (36)	33 (42)	
Size	< 5 cm	30 (46)	9 (14)	0.1110	27 (41)	12 (18)	**0.0413**	25 (38)	14 (22)	**0.0021**	21 (33)	17 (27)	0.1302	24 (38)	14 (22)	**0.0409**
	≥ 5 cm	15 (23)	11 (16)		11 (17)	15 (23)		6 (9)	20 (31)		9 (14)	17 (27)		9 (14)	17 (27)	
	N/A															
Pathological fracture	Absent	46 (62)	22 (29)	0.1701	39 (52)	29 (39)	0.3910	33 (45)	35 (47)	0.2003	31 (42)	36 (49)	1.0000	33 (45)	34 (47)	1.0000
	Present	6 (8)	0 (0)		5 (7)	1 (1)		5 (6)	1 (1)		3 (4)	3 (4)		3 (4)	3 (4)	
Neoadjuvant denosumab	Absent	65 (70)	21 (23)	**0.0064**	54 (59)	32 (35)	0.2051	44 (48)	42 (46)	1.0000	42 (47)	43 (48)	1.0000	43 (48)	42 (47)	1.0000
	Present	1 (1)	5 (5)		2 (2)	4 (4)		3 (3)	3 (3)		2 (2)	3 (3)		3 (3)	2 (2)	
Local recurrence	Absent	43 (57)	12 (16)	**0.0457**	33 (43)	22 (29)	0.6086	34 (45)	21 (28)	**0.0389**	24 (32)	31 (41)	0.1227	30 (40)	25 (33)	0.3049
	Present	11 (14)	10 (13)		11 (14)	10 (13)		7 (9)	14 (18)		13 (17)	7 (9)		8 (11)	12 (16)	
Distant metastasis	Absent	50 (66)	19 (25)	0.4059	41 (54)	28 (37)	0.4456	39 (51)	30 (39)	0.2375	35 (47)	33 (44)	0.4303	35 (47)	33 (44)	0.7110
	Present	4 (5)	3 (4)		3 (4)	4 (5)		2 (3)	5 (6)		2 (3)	5 (7)		3 (4)	4 (5)	
**Pathological and genetic feature**																
Mitosis 10/10 HPFs	1–9	53 (59)	21 (23)	1.0000	47 (52)	27 (30)	0.1672	41 (46)	33 (37)	0.0513	40 (45)	33 (37)	**0.0121**	38 (43)	35 (33)	0.5912
	≥ 10	12 (13)	4 (4)		7 (8)	9 (10)		4 (4)	12 (13)		3 (3)	13 (15)		7 (8)	9 (10)	
Osteoclastic multinucleated giant cell	Low	31 (34)	13 (14)	0.8152	29 (32)	15 (17)	0.2890	19 (21)	25 (28)	0.2917	21 (24)	22 (25)	1.0000	20 (22)	23 (26)	0.5272
	High	34 (38)	12 (13)		25 (28)	21 (23)		26 (29)	20 (22)		22 (25)	24 (27)		25 (28)	21 (23)	
Spindle cell feature	Absent	39 (43)	13 (14)	0.6343	35 (39)	17 (19)	0.1282	32 (36)	20 (22)	**0.0184**	29 (33)	22 (25)	0.0862	29 (33)	22 (25)	0.2016
	Present	26 (29)	12 (13)		19 (21)	19 (21)		13 (14)	25 (28)		14 (16)	24 (27)		16 (18)	22 (25)	
Foamy macrophage	Absent	57 (63)	22 (24)	1.0000	50 (56)	29 (32)	0.1081	41 (46)	38 (42)	0.5216	38 (43)	40 (45)	1.0000	43 (48)	35 (39)	**0.0266**
	Present	8 (9)	3 (3)		4 (4)	7 (8)		4 (4)	7 (8)		5 (6)	6 (7)		2 (2)	9 (10)	
Osteoid formation	Absent	31 (34)	8 (9)	0.2366	24 (27)	15 (17)	0.8310	22 (24)	17 (18)	0.3950	21 (24)	18 (20)	0.3977	21 (24)	18 (20)	0.6708
	Present	34 (38)	17 (19)		30 (33)	21 (23)		23 (26)	28 (31)		22 (25)	28 (31)		24 (27)	26 (29)	
Secondary aneurysmal bone cystic change	Absent	52 (58)	20 (22)	1.0000	42 (47)	28 (32)	0.7871	36 (40)	36 (40)	1.0000	35 (39)	37 (42)	1.0000	40 (45)	32 (36)	0.0631
	Present	13 (14)	5 (6)		12 (13)	6 (7)		9 (10)	9 (10)		8 (9)	9 (10)		5 (6)	12 (13)	
H3F3A mutation	G34W	62 (67)	24 (26)	1.0000	51 (55)	35 (38)	0.3979	43 (48)	42 (47)	0.7837	44 (48)	42 (46)	1.0000	43 (48)	42 (47)	1.0000
	Others	4 (4)	2 (2)		5 (5)	1 (1)		1 (1)	4 (4)		3 (3)	3 (3)		3 (3)	2 (2)	

Bold value indicates significant differences.

PD-L1; programmed death ligand-1. IDO-1: indoleamine 2,3-dioxygenase-1. SIRPα: signal-regulatory protein α. FOXP3: forkhead box P3. CD8: cluster of differentiation 8.

##### Association of PD-L1 and IDO1 expression with SIRPα^+^ macrophage and FOXP3^+^ and CD8^+^ lymphocyte infiltration

**Figure 2:**
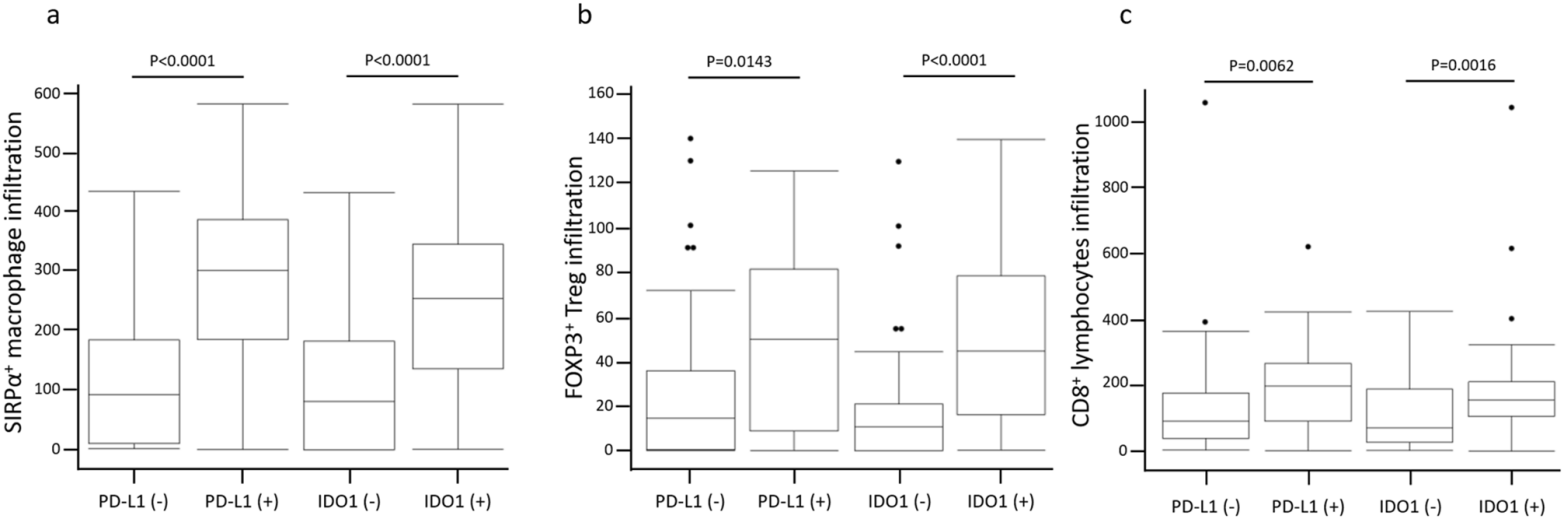
The correlations of infiltration of SIRPα (**a**), FOXP3 (**b**) and CD8 (**c**) positive cells with the immunoexpression of PD-L1 and IDO1.

We evaluated the correlations between PD-L1 and IDO1 expression and SIRPα^+^, FOXP3^+^, and CD8^+^ infiltration. There was significantly more SIRPα^+^, FOXP3^+^, and CD8^+^ infiltration in all specimens from PD-L1-positive patients than from PD-L1-negative patients (*P* < 0.0001, *P* = 0.0143, and *P* = 0.0062, respectively) (Fig. 2a–c). In all specimens with IDO1 positivity, the infiltration of SIRPα^+^, FOXP3^+^, and CD8^+^ cells was significantly higher than in equivalent IDO1-negative cases (*P* < 0.0001, *P* < 0.0001, and *P* = 0.0016, respectively) (Fig. 2a-c).

#### Alteration of PD-L1 and IDO1 expression and SIRPα^+^ macrophage and FOXP3^+^ and CD8^+^ lymphocyte infiltration after denosumab treatment

##### Differences in PD-L1 and IDO1 expression between primary, ND-Rec, and D-Rec lesions

**Figure 3:**
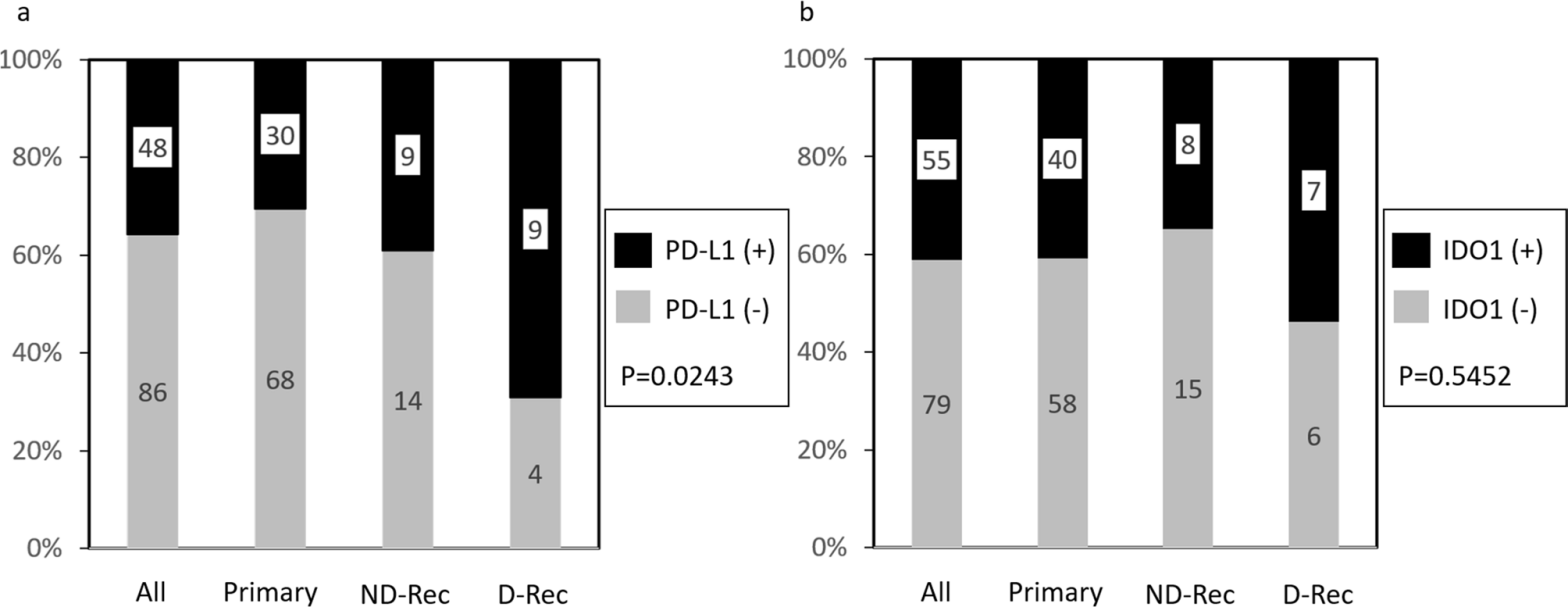
The changes of PD-L1 (**a**) and IDO1 (**b**) immunoexpression between primary lesion, ND-Rec and D-Rec. D-Rec: Recurrent lesion which treated by denosumab ND-Rec: Recurrent lesion which not treated by denosumab.

We evaluated PD-L1 and IDO1 expression in primary lesions, recurrent lesions after denosumab treatment (D-Rec), and recurrent lesions not treated with denosumab (ND-Rec). The frequency of PD-L1 expression in D-Rec lesions was significantly higher than in primary and ND-Rec lesions (*P* = 0.0243) (Fig. 3a). There were no significant differences in IDO1 expression between primary, ND-Rec, and D-Rec lesions (Fig. 3b).

##### Differences in SIRPα, FOXP3, and CD8 infiltration between primary, ND-Rec, and D-Rec lesions

**Figure 4:**
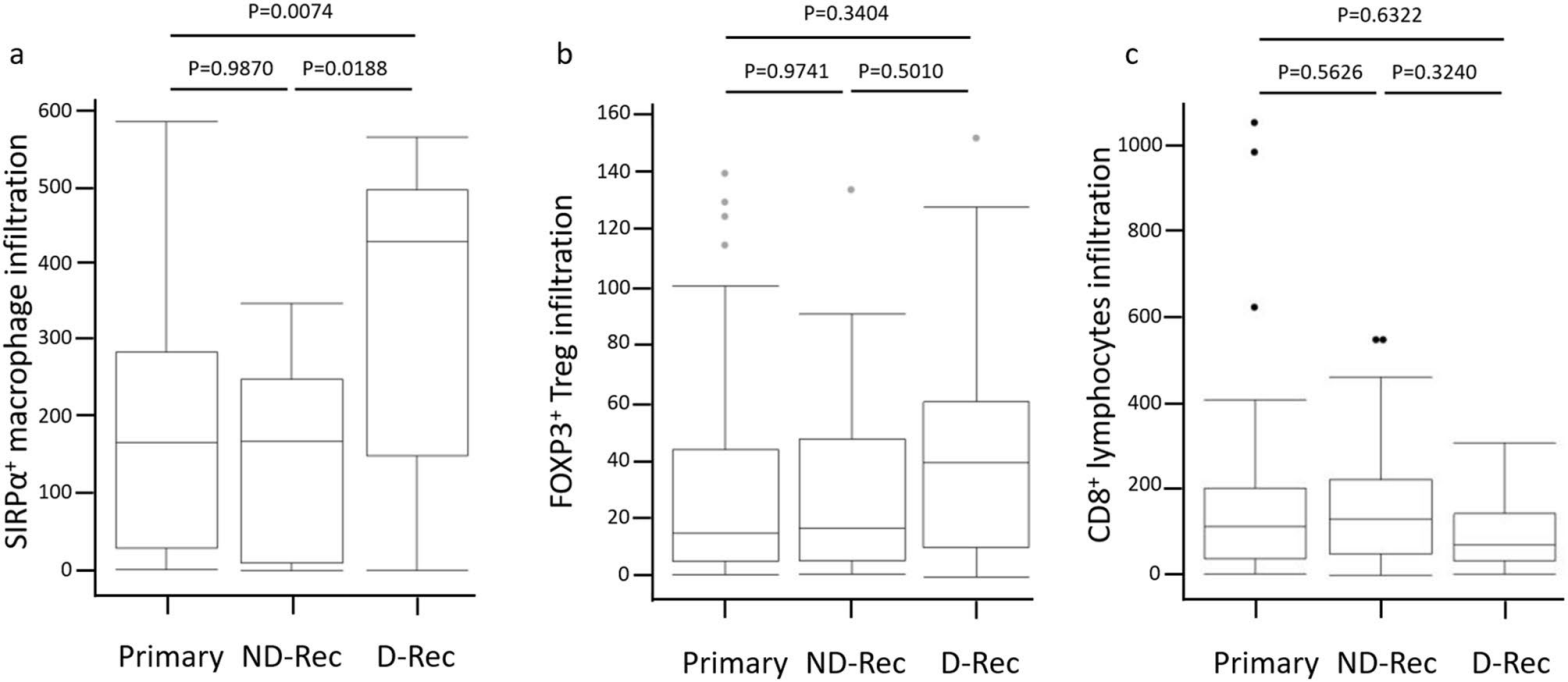
The changes of infiltration of SIRPα (**a**), FOXP3 (**b**) and CD8 (**c**) positive cells between primary lesion, ND-Rec and D-Rec.

**Figure 5:**
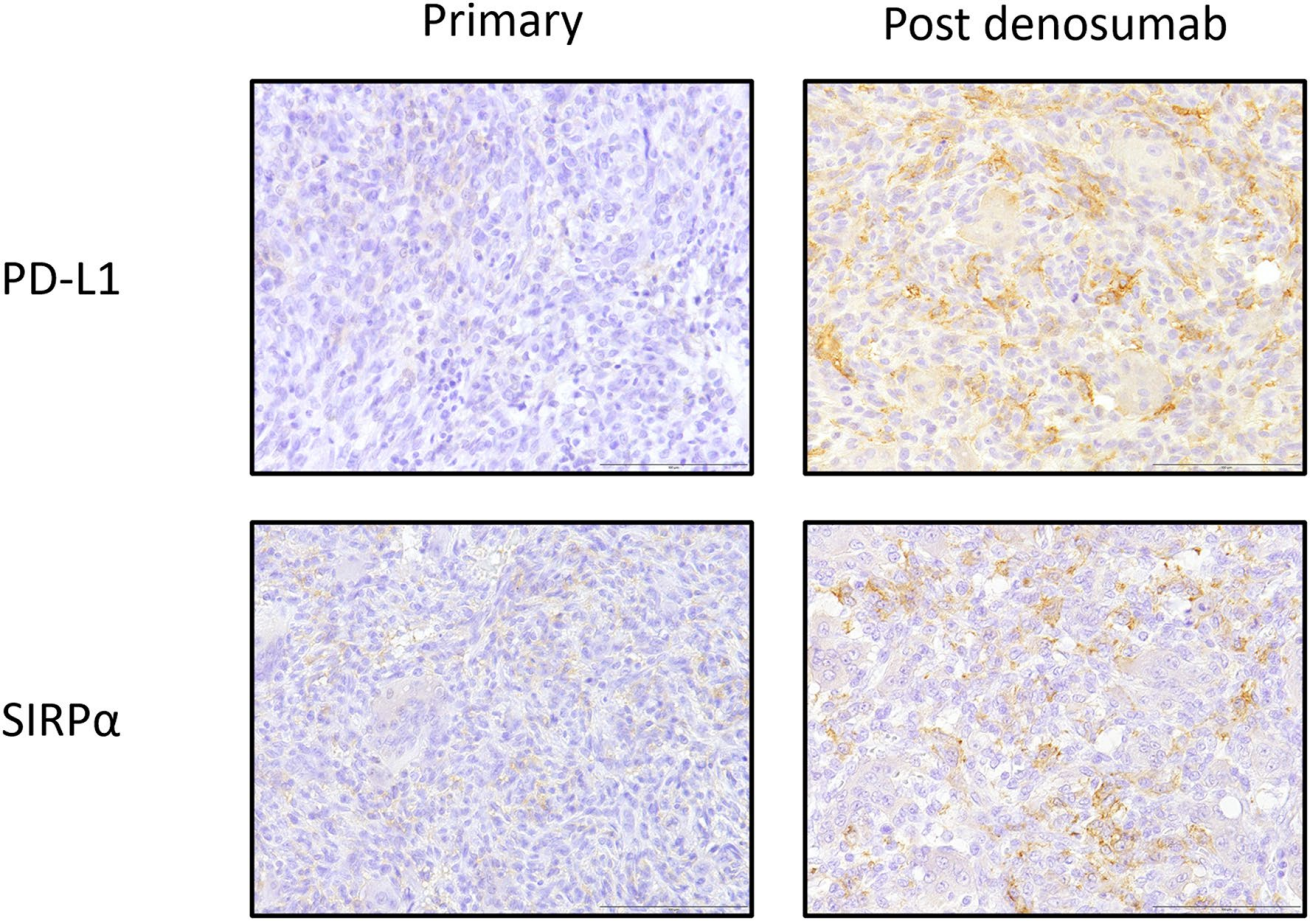
Representative figures of PD-L1 and SIRPα that compared pre- and post- denosumab treatment belong to same patient. Scale bars: 100 µm.

We compared SIRPα^+^, FOXP3^+^, and CD8^+^ cell counts between primary, ND-Rec, and D-Rec lesions. SIRPα^+^ cell infiltration in D-Rec lesions was significantly increased compared with that in primary lesions and ND-Rec lesions (*P* = 0.0074 and *P* = 0.0188, respectively) (Fig. 4a). Representative figures of PD-L1 and SIRPα that compared pre- and post- denosumab treatment belong to same patient were shown in Fig. 5. There was no significant difference in SIRPα^+^ cell infiltration between primary tumors and ND-Rec lesions. FOXP3 and CD8 infiltration in primary, ND-Rec, and D-Rec lesions did not reach statistical significance (Fig. 4b,c).

#### Prognostic significance of PD-L1 and IDO1 expression, tumor-infiltrating lymphocytes (FOXP3 and CD8), SIRPα infiltration, and clinico-radio-pathological features in primary naive lesions

**Figure 6:**
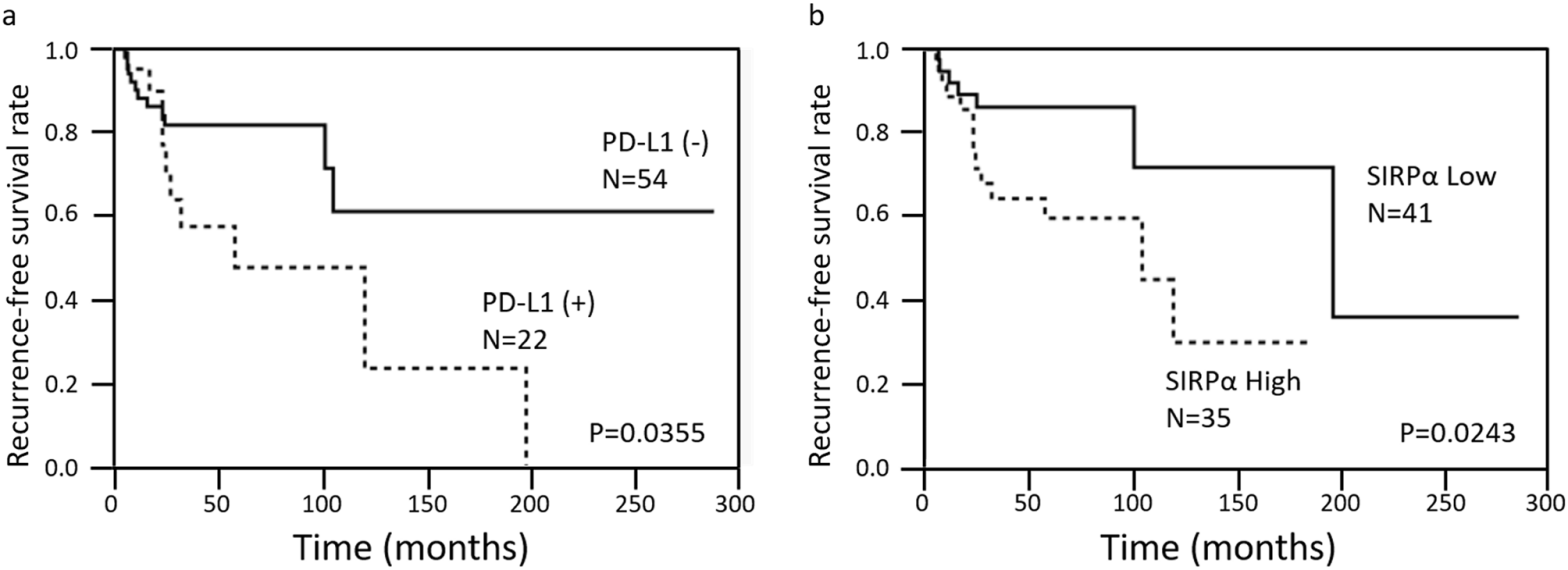
Kaplan–Meier survival curves of PD-L1 expression (**a**) and infiltration of SIRPα (**b**) illustrate recurrence-free survival (log-rank test).

We assessed the prognostic significance of clinico-radio-pathological characteristics, PD-L1 and IDO1 expression, and SIRPα^+^, FOXP3^+^ and CD8^+^ cell infiltration by using Kaplan–Meier survival analysis (Table 3). PD-L1 expression and a high number of SIRPα^+^ cells were correlated with shorter recurrence-free survival (*P* = 0.0355 and *P* = 0.0243, respectively) (Table 3, Fig. 6a,b). There was no significant correlation between recurrence-free survival and preoperative denosumab treatment. The results of multivariate analysis of clinicopathological features and immunohistochemical studies suggested that high SIRPα^+^ and FOXP3^+^ infiltration were associated with shorter recurrence-free survival (Table 3).

**Table 3:** Univariate and multivariate for recurrence-free survival.

Factors		5 yr R-FS(%)	Univariate	Multivariate		
			*P* value	Odd ratio	95%CI	*P* value
Age(year)	< 33	66.7	0.3819	2.25	0.69–7.33	0.1764
	≥ 33	76.8				
Gender	Male	77.2	0.7038			
	Female	68.5				
Tumor site	Extremity	71.3	0.9502			
	Axial skeleton	77.1				
Extremity	Upper extremity	79.1	0.5217			
	Lower extremity	69.2				
Size	< 5 cm	65.1	0.3700	2.56	0.79–8.24	0.1163
	≥ 5 cm	80.7				
Pathological features	Absent	70.6	0.6917			
	Present	75.0				
Neoadjuvant denosumab therapy	Given	*	0.2618			
	No given	73.8				
H3F3A mutataion	G34W	73.6	0.4611			
	G34R or G34V	75.0				
Mitosis(/10 HPFs)	1–9	76.2	0.4773			
	≥ 10	64.8				
Osteoclastic-giant cell	High	75.8	0.8776			
	Low	70.4				
Foamy macrophages	Present	100.0	0.7734			
	Absent	70.9				
Bone formation	Present	76.3	0.6443			
	Absent	67.4				
Spindle cell feature	Present	64.9	0.2527			
	Absent	79.9				
Secondary aneurysmal bone cystic change	Present	69.3	0.3256			
	Absent	77.5				
PD-L1	Positive	48.1	**0.0355**	0.92	0.28–3.03	0.8947
	Negative	81.6				
IDO1	Positive	69.8	0.8796			
	Negative	76.1				
SIRPα	High	59.6	**0.0243**	7.07	1.66–30.2	**0.0083**
	Low	86.1				
FOXP3	High	80.0	0.1190	0.26	0.08–0.87	**0.0285**
	Low	68.7				
CD8	High	69.4	0.4827			
	Low	79.2				

Bold value indicates significant differences.

* There was no case with more than 5 years follow-up.

PD-L1; programmed death ligand-1, IDO-1: indoleamine 2,3-dioxygenase-1, SIRPα: signal-regulatory protein α, FOXP3: forkhead box P3, CD8: cluster of differentiation 8, R-S survival; Recurence-free survival.

### Immunofluorescence double staining

#### Localization of PD-L1 expression in the tumor microenvironment of GCTB

**Figure 7:**
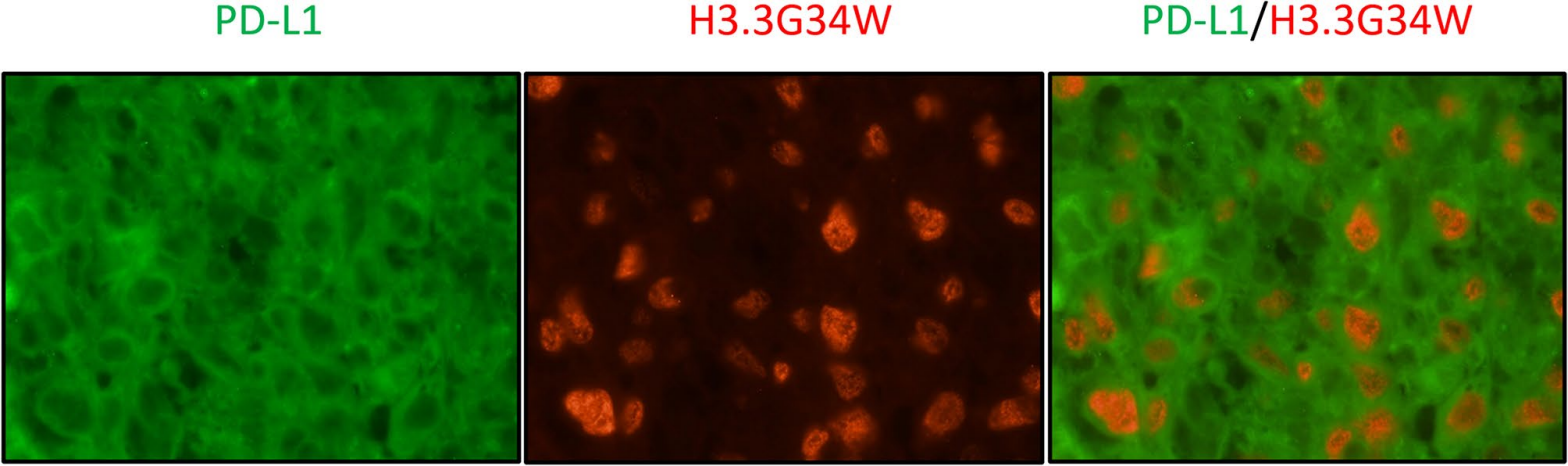
Double immunofluorescence stain showed the tumor cells with H3.3G34W mutation and mononuclear stroma cells had PD-L1 expression.

We performed double immunofluorescence (n = 10) to evaluate whether tumor cells with *H3F3A* mutations or mononuclear histiocytoid cells express PD-L1. Representative images are shown in Fig. 7. Both tumor cells with *H3F3A* mutations and mononuclear histiocytoid cells were found to express PD-L1.

#### Localization of SIRPα on mononuclear cells of GCTB

**Figure 8:**
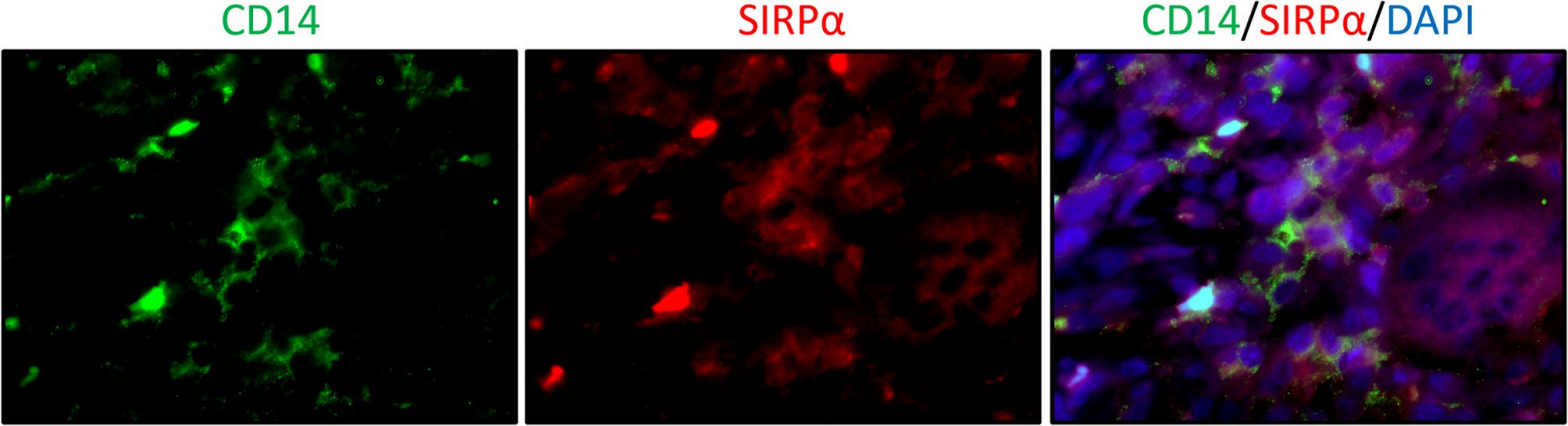
Double immunofluorescence stain showed some of SIRPα-positive cells (red) expressed CD14 (green).

Moreover, to verify which cells express SIRPα, double immunofluorescence was performed for SIRPα and CD14 (n = 10). Representative images are shown jn Fig. 8. SIRPα positive cells were diffusely found and some of these positive cells also expressed CD14 (monocyte marker).

## Discussion

Classically, GCTB is treated with chemical adjuvant and/or surgery to reduce local recurrence. However, the rate of local recurrence after chemical adjuvant therapy is 15–50%^2,14^. Local recurrence significantly debases patients’ activities of daily living and quality of life. Thus, prevention of local recurrence is an important part of GCTB treatment. Denosumab, which is a human monoclonal anti-RANKL antibody that inhibits the RANK/RANKL signaling pathway, has been introduced to treat GCTB^12,13,34^. Pathological findings have previously revealed osteoclast-like multinucleated giant cell and mononuclear stromal cell depletion and new bone formation after denosumab treatment^11,35,36^. GCTB sometimes shows dramatic histological changes and mimics primary malignant bone tumors after denosumab treatment^35,36^. Kato et al. reported that after denosumab therapy, mononuclear tumor cells with *H3F3A* mutations could survive, but osteoclast cells could not survive without RANK/RANKL signaling^6^. Some reports have recently suggested that preoperative denosumab administration might increase the recurrence rate after operation^34,37^. New bone formation may be difficult to distinguish from preexisting bone trabeculae and make it difficult to identify true surgical margins^34^. No consensus has been reached about whether preoperative denosumab treatment might be useful to prevent local recurrence. Some GCTB cases are refractory to denosumab and have frequent recurrence. Thus, novel therapeutic targets, such as immunotherapy against GCTB, are required.

There have been few studies concerning tumor immunity in GCTB and immune microenvironment alterations after denosumab therapy. To understand the tumor microenvironment of GCTB, we established this study to focus on the tumor immune microenvironment of primary lesions and recurrent lesions with and without denosumab therapy in GCTB patients, and to investigate an immune checkpoint inhibitor treatment strategy for GCTB patients. A prior immunohistochemical study showed PD-L1 expression by tumor cells and multinucleated giant cells in 28.3% of GCTB specimens, and this was associated with shortened disease-free survival and high Ki-67 positivity^22^. Moreover, this previous study focused on two immune-related genes, *TLR8* and *LCK*, which are related to innate immunity activation and CD4^+^ and CD8^+^ lymphocyte development. Lower *TLR8* and *LCK* expression were correlated with PD-L1 immunoexpression positivity, but after denosumab treatment, *TLR8* and *LCK* expression increased^22^. Our results showed PD-L1 immunoexpression in about 28% of primary specimens without denosumab treatment, and this was significantly related to shortened recurrence-free survival. Our double immunofluorescence staining study showed that PD-L1 expression in both neoplastic cells with *H3F3A* mutations and in mononuclear histiocytoid cells were seen. Moreover, the frequency of PD-L1 in D-Rec lesions was significantly higher than in primary and ND-Rec lesions. These results suggested possible interactions between tumor cells and mononuclear histiocytoid cells via the PD-1/PD-L1 axis in addition to RANK/RANKL signaling. It is well known that in GCTB, RANK/RANKL axis between neoplastic cells and mononuclear histiocytoid cells mediates osteoclastic pathways^6^. On the other hand, in tumor microenvironment, PD-1/PD-L1 axis enable tumor cells to avoid immune surveillance^16–21^. Our results suggested that tumor cells with *H3F3A* mutation and mononuclear histiocytoid cells might cooperate to express PD-L1 and escape tumor immunity because neoplastic cells and non-neoplastic cells expressed PD-L1. Moreover, our double immunofluorescence staining showed that SIRPα positive cells were diffusely found and some of these positive cells also expressed CD14 (monocyte marker). In GCTB, histologically mononuclear cells are tumor cells or monocytes^11^. Therefore, we suggested that tumor cells also would express SIRPα, although double staining for H3G34W and SIRPα was not possible in our study. There was the possibility of immune alterations associated with recurrence and denosumab treatment. More specifically, PD-L1 and SIRPα were more upregulated in recurrent tumors than in primary tumors due to resistance to treatment. It is suggested that tumor cells and/or non-neoplastic cells escaped tumor immunity by expressing PD-L1 and SIRPα.

Thus, patients with uncontrollable recurrent lesions may be treated with anti-PD-L1 or anti-PD-1 inhibitors after denosumab therapy.

On the other hand, the use of widely variable combinations of immune checkpoint inhibitors has gained great interest to improve immunotherapy outcomes^31^. For instance, macrophage-related immune checkpoints and CD47/SIRPα interactions have been focused on as new therapeutic targets of immunotherapy^31,32,38,39^. We showed that high SIRPα^+^ infiltration in primary specimens was associated with shorter recurrence-free survival by using univariate and Cox multivariate analyses. SIRPα^+^ macrophages were frequently seen in recurrent specimens treated with denosumab rather than in primary specimens with frequent PD-L1 immunopositivity. Previous studies have reported that SIRPα^+^ macrophage infiltrates were correlated with shorter overall survival and progression-free survival in other malignancies, such as diffuse large B-cell lymphoma^32^ and melanoma and renal cell carcinoma^39^. However, other reports have also shown a positive correlation between SIRPα expression and good prognosis^33^. In our immunohistochemical studies, it was suggested that SIRPα enabled tumor cells to evade phagocytosis, leading to promoted tumor growth and progression, similar to PD-L1 expression in GCTB patients. Treatment inhibiting CD47 and SIRP interactions has been investigated in previous studies, with clinical trials in hematopoietic and solid cancers^40–42^. We suggest that anti-SIRPα inhibitors will become a treatment for GCTB patients, especially patients with frequent recurrence.

Several studies have reported that RANK/RANKL inhibitors have the potential to increase the effectiveness of immune checkpoint inhibitors^43–48^. In a mouse study, Ahern et al. showed that combination therapy with anti-cytotoxic T-lymphocyte-associated protein 4 (CTLA-4) inhibitors and anti-RANKL inhibitors increased CD8-positive T-cell infiltration compared to anti-CTLA-4 inhibitors or anti-RANKL inhibitors alone^46^. Our immunohistochemical study results showed no significant differences in CD8 infiltration between primary, ND-Rec, and D-Rec specimens. However, it was suggested that in GCTB, CD8^+^ lymphocyte infiltration would increase with combined immunotherapy and denosumab therapy. In a previous study, patients with lung cancer or melanoma who received both anti-CTLA-4 and/or anti-PD-1 inhibitors and denosumab had good disease control rates compared to patients not receiving denosumab^48^. Moreover, the authors of this previous study claimed that longer use of combined therapy was preferable to control tumor progression^48^. Although denosumab is widely used for GCTB treatment, the potential of recurrence remains. In our study, PD-L1 and SIRP expression in recurrent GCTB with denosumab treatment was higher than in primary GCTB and recurrent GCTB without denosumab treatment. Our findings suggested that the combination of PD-L1/PD-1 and/or SIRP inhibitors and denosumab might be effective for controlling recurrent GCTB. Further studies using a larger number of cases are required to confirm the effectiveness of these treatments. To the best of our knowledge, this study is the first to report SIRP expression in GCTB and investigate alterations in primary specimens and recurrent specimens with and without denosumab treatment. From the immunohistochemical and immunofluorescence results in this study, it can be concluded that PD-L1 and SIRPα immune checkpoint inhibitors may provide clinical benefits in GCTB patients with uncontrollable recurrent lesions after denosumab therapy.

## Materials and methods

### Patients and tissue samples

We used samples of GCTB patients registered from 1984 to 2019 in the database of the Department of Anatomic Pathology, Graduate School of Medical Sciences, Kyushu University, Fukuoka, Japan. All cases were reviewed based on histological examinations with hematoxylin and eosin staining and immunohistochemical studies using antibodies specific to GCTB (anti-H3.3G34W, anti-H3.3G34R, and anti-H3.3G34V)^4,7,11^.

This study included 137 formalin-fixed, paraffin-embedded samples from 96 patients. The samples included primary conventional GCTB, recurrent conventional GCTB, post-denosumab GCTB, and lung metastasis of conventional GCTB. The lesions were collected by biopsy or resection. Clinical data, including age at diagnosis, sex, and tumor site, were collected. The presence of pathological fractures was investigated via plain radiographs, computed tomography scans, and/or magnetic resonance imaging scans that were examined by a radiologist (J.M.). Morphological features, mitotic figures, osteoclastic giant cells, foamy macrophages, bone formation, spindle cell features, and secondary aneurysmal bone cystic changes were also evaluated and investigated by three pathologists (Y.T., K.K., and Y.Y.). The institutional review board at Kyushu University approved this study (approval codes: 29-625 and 29-429). Written informed consent was obtained from the patients and their parents/guardians prior to tissue collection. All experiments were performed in accordance with guidelines provided by the Ethics Committees and Institutional Review Boards.

### Immunohistochemical staining

For the immunohistochemical and immunofluorescence studies, formalin-fixed, paraffin-embedded tissues were sliced into 4-μm sections. The immunohistochemical studies were performed as previously described^20,29^. The following rabbit and mouse monoclonal antibodies were used as the primary antibodies: anti-PD-L1, anti-IDO1, anti- SIRPα, anti-FOXP3, anti-CD8, anti-H3.3G34W, anti-H3.3G34R, and anti-H3.3G34V (Supplementary Table S1). Appropriate controls were used throughout. Three pathologists (Y.T., K.K., and Y.Y.) independently evaluated the immunohistochemical staining results for each sample. For the immunohistochemical evaluation of PD-L1 and IDO1, the membrane PD-L1 expression and cytoplasmic IDO1 expression were defined by combined proportion scores, which evaluates on tumor cells and tumor-associated immune cells. Cases with a combined proportion score ≥ 1% were defined as positive. Moreover, SIRPα^+^, CD8^+^, and FOXP3^+^ cells were counted per high power field in five dependent fields for each case. This process was based on previous studies, with modifications^32,33,49–51^. Statistically, the median numbers of SIRPα^+^, CD8^+^, and FOXP3^+^ cells were determined as the cut-off points.

### Immunofluorescence double staining

To identify PD-L1 and H3.3G34W localization in mononuclear stromal cells and tumor cells, double immunofluorescence was performed for H3.3G34W and PD-L1 (n = 10). Moreover, to verify which cells express SIRPα, double immunofluorescence was performed for SIRPα and CD14 (n = 10). The following antibodies were used as the primary antibodies: anti-PD-L1, anti-H3.3G34W, anti-SIRPα and anti-CD14 (Supplementary Table S1).

### Statistical analysis

Fisher’s exact or Wilcoxon tests were used to analyze correlations between two dichotomous variables, such as clinicopathological findings and immunohistochemical results. Survival curves were calculated by using the Kaplan–Meier method, and significant differences were calculated by using the log-rank test. Statistical significance was defined as *P* < 0.05. In the multivariate analysis, a Cox proportional hazards model was used. Data analysis was performed by using the JMP statistical software package (version JMP version 14.2.0; SAS Institute Inc., Cary, NC, USA).

## Supplementary Information

Supplementary Information 1.

## Data Availability

All data generated or analysed during this study are included in this published article and its Supplementary Information files.
