# Supplementary figures and images for: Tumor microenvironment in giant cell tumor of bone: evaluation of PD-L1 expression and SIRPα infiltration after denosumab treatment
Source: Sci Rep. 2021 Jul 20;11:14821. doi: 10.1038/s41598-021-94022-w (PMC8292371; doi:10.1038/s41598-021-94022-w)

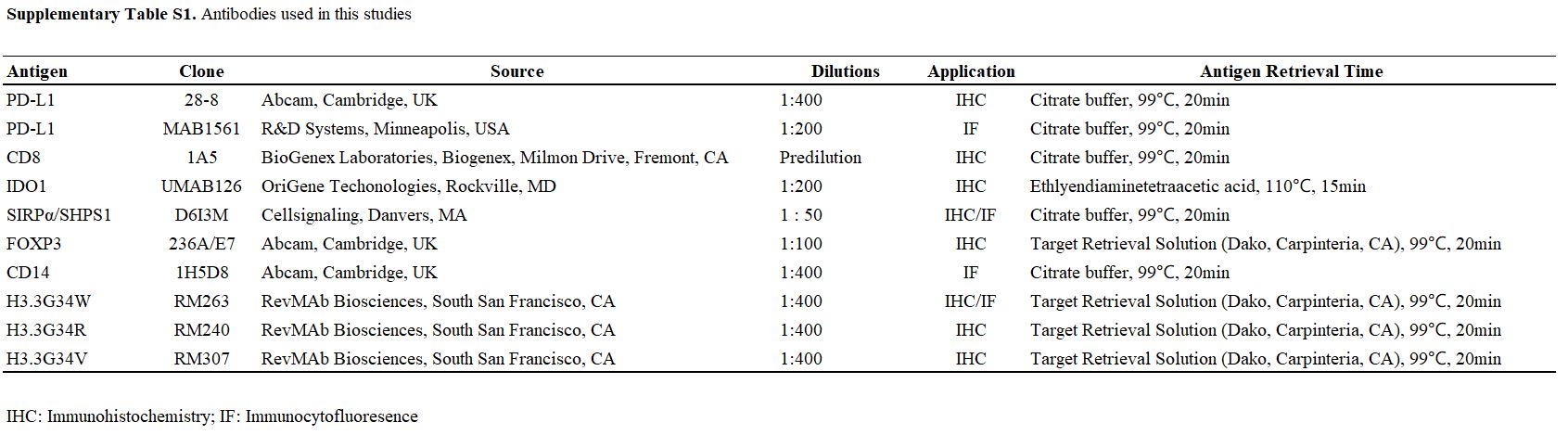

Supplement: Supplementary file 1 — Supplementary Information 1. [file 41598_2021_94022_MOESM1_ESM.jpg]
